# Zika virus NS3 is a canonical RNA helicase stimulated by NS5 RNA polymerase

**DOI:** 10.1093/nar/gkz650

**Published:** 2019-07-30

**Authors:** Shan Xu, Yali Ci, Leijie Wang, Yang Yang, Leiliang Zhang, Caimin Xu, Chengfeng Qin, Lei Shi

**Affiliations:** 1 State Key Laboratory of Medical Molecular Biology, Institute of Basic Medical Sciences, Chinese Academy of Medical Sciences and School of Basic Medicine, Peking Union Medical College, Beijing 100005, China; 2 Department of Biochemistry and Molecular Biology, Institute of Basic Medical Sciences, Chinese Academy of Medical Sciences and School of Basic Medicine, Peking Union Medical College, Beijing 100005, China; 3 Institute of Pathogen Biology, Chinese Academy of Medical Sciences, Beijing, China; 4 State Key Laboratory of Pathogen and Biosecurity, Beijing Institute of Microbiology and Epidemiology, Beijing 100071, China

## Abstract

Zika virus is a positive single-strand RNA virus whose replication involved RNA unwinding and synthesis. ZIKV NS3 contains a helicase domain, but its enzymatic activity is not fully characterized. Here, we established a dsRNA unwinding assay based on the FRET effect to study the helicase activity of ZIKV NS3, which provided kinetic information in real time. We found that ZIKV NS3 specifically unwound dsRNA/dsDNA with a 3′ overhang in the 3′ to 5′ direction. The RNA unwinding ability of NS3 significantly decreased when the duplex was longer than 18 base pairs. The helicase activity of NS3 depends on ATP hydrolysis and binding to RNA. Mutations in the ATP binding region or the RNA binding region of NS3 impair its helicase activity, thus blocking viral replication in the cell. Furthermore, we showed that ZIKV NS5 interacted with NS3 and stimulated its helicase activity. Disrupting NS3-NS5 interaction resulted in a defect in viral replication, revealing the tight coupling of RNA unwinding and synthesis. We suggest that NS3 helicase activity is stimulated by NS5; thus, viral replication can be carried out efficiently. Our work provides a molecular mechanism of ZIKV NS3 unwinding and novel insights into ZIKV replication.

## INTRODUCTION

Flaviviruses are positive single-strand RNA viruses that include Dengue virus (DENV), Yellow Fever virus (YFV), West Nile virus (WNV), Japanese Encephalitis virus (JEV), Zika virus (ZIKV) as well as some other viruses. As an emergent threat to humans, outbreak of ZIKV in 2016 caused serious concern worldwide (1). Epidemiological and biological studies showed that ZIKV infection is strongly associated with neonatal microcephaly and with Guillain-Barré syndrome in adults (2–4). Unfortunately, there is not yet any effective treatment for ZIKV infection (5). Previous studies on ZIKV provided only limited information about its replication in the cell, and the detailed mechanisms are still unclear. Therefore, it is of great importance to explore the replication process of ZIKV, which may offer new strategies for ZIKV prevention and therapy.

Similar to other flavivirus family members, ZIKV genomic RNA is approximately 11kb in length, and encodes three structural proteins (C, prM/M and E protein) and seven nonstructural proteins (NS1, NS2A, NS2B, NS3, NS4A, NS4B and NS5) (6). The structural proteins are the components for assembling viral particles, and the nonstructural proteins perform viral replication in the cell. Two viral proteins, NS3 and NS5, possess all enzymatic functions and dominate viral RNA amplification (7,8). The viral RNA replication process is described briefly as below (9). First, NS5, the RNA-dependent RNA polymerase (RdRp), synthesizes a negative-sense RNA using the positive-sense genomic RNA as the template, resulting in a dsRNA intermediate, which is unwound by NS3 to generate separate negative-sense and positive-sense RNAs. The negative-sense RNA serves as a new template for the production of substantial amounts of positive-sense genomic RNA. After 5′ capping and methylation with the cooperation of NS3 and NS5, the positive-sense RNA is mature and assembled into viral particles.

Except for the first round of RNA synthesis, which directly uses viral genomic (+) RNA as a template, the opening of the dsRNA intermediate by NS3 is the prerequisite step for all the remaining steps in viral RNA synthesis. Undoubtedly, NS3 plays a crucial role in viral replication. Flaviviral NS3 has two structurally separated functional domains, the N-terminal protease domain and the C-terminal helicase domain (10). The N-terminal protease cleaves the viral single-chain polyprotein precursor into individual proteins, and it also cleaves some host factors (11–16). The C-terminal helicase is responsible for dsRNA unwinding during viral RNA synthesis. Although previous biochemical studies revealed the helicase activity of flaviviral NS3 using traditional electrophoretic mobility shift assay (EMSA), real-time kinetic information about dsRNA unwinding is lacking due to the limited temporal resolution of the technique (17,18). In addition, the lack of studies combining biochemical analysis of enzymatic activity and functional assays of viral genomic amplification restricts our mechanistic understanding of viral replication in the cell (19). Moreover, unlike the NS3 from other flavivirus members, studies on ZIKV NS3 are still rare. Thus, characterization of ZIKV NS3 enzymatic activity and its related function during viral genome amplification are important issues for understanding the mechanism of ZIKV replication.

Here, we establish a dsRNA/DNA unwinding assay based on fluorescence resonance energy transfer (FRET) to monitor ZIKV NS3 helicase activity in real time. We show that ZIKV NS3 has RNA and DNA helicase activity depending on ATP hydrolysis, and it preferentially unwinds RNA/DNA duplexes with a 3′ overhang in vitro. Several residues (G199, K200, D410 and K431) residing in the ATP binding site and the RNA binding site of NS3 are crucial for its helicase activity, and mutations at these sites abolish viral replication and production. Notably, we found that NS5 stimulates NS3 helicase activity through increasing its dsRNA unwinding velocity. This effect depends on the interaction between NS3 and NS5. Interrupting this interaction loses the stimulation of NS3 helicase activity by NS5 *in vitro* and leads to significant deficiencies in ZIKV replication. Our work systematically characterizes ZIKV NS3 helicase activity and suggests cooperation between NS3 and NS5 in viral replication, shedding light on the mechanism that couples ZIKV RNA unwinding and synthesis.

## MATERIALS AND METHODS

### Cell lines and antibodies

BHK-21 cells were maintained in DMEM medium (Hyclone) supplemented with 5% fetal bovine serum (FBS) at 37°C with 5% CO_2_. Vero cells were grown in DMEM medium (Hyclone) containing 10% FBS at 37°C with 5% CO_2_. The following antibodies were used: mouse monoclonal antibody (mAb) J2 anti-double strand RNA (dsRNA) (Scicons, cat# J2-1406), mouse mAb anti-zika Envelope protein (Biofront, cat# BF-1176-56), and goat anti-mouse IgG conjugated with Alexa Fluor 488 (Invitrogen, cat# A28175).

### Clone construction

ZIKV NS3 or NS5 sequence was amplified using gene specific primers and then inserted into the indicated vector using restriction enzyme digestion and ligation method. NS3 mutants were generated by site-directed mutagenesis according to Quik*Change*^®^/*I* Site-Directed Mutagenesis manual. All mutations were confirmed by sequencing.

### Expression and purification of recombinant proteins

All recombinant proteins were expressed in bacteria. In brief, *Escherichia coli* BL21 cells, transformed with ZIKV NS3-pET28 or NS5-pET28 plasmids, were cultured at 37°C in Luria-Bertani medium containing kanamycin (100 ug/ml) until the optical density at 600 nm reached 0.8. The cells were induced with 0.4 mM isopropyl-b-d-thiogalactopyranoside (IPTG) at 16°C for 24 h. Bacterial were harvested, then resuspended and sonicated in lysis buffer (500 mM NaCl, 5 mM MgCl_2_, 20 mM Tris–HCl pH 8.0, 0.5% TritonX-100, 20 mg/l RNaseA, 20 mg/l DNaseI, 10% glycerol, 1 mM DTT with proteinase inhibitors) on ice. The cell lysate was centrifuged at 18 000 rpm at 4°C for 45 min. His-tagged protein was purified from the supernatant with Ni-NTA Agarose beads according to the manual. The eluate was dialyzed with buffer containing 100 mM NaCl, 20 mM Tris–HCl pH 8.0, 10% glycerol and 1 mM DTT. NS5 was further purified by gel filtration chromatography using a Superdex200 10/300GL (GE Healthcare) with a running buffer of 20 mM Tris–HCl, 500 mM NaCl pH 8.0. The collected protein fractions were concentrated to 3 mg/ml by using a membrane concentrator with a molecular weight cutoff of 30 kDa (Millipore).

### ATPase activity assay

The ATPase activity was detected with QuantiChrom™ATPase/GTPase Assay Kit (BioAssay Systems). The free phosphate released by ATP hydrolysis was quantified with the reagent in the kit to indicate the ATPase activity. Phosphate standards were prepared according to the manual. The reaction was performed in a final volume of 40 μl in 96-well plates. 30 nM wild type or mutated ZIKA NS3 protein were incubated with various concentrations of ATP in buffer (20 mM Tris, 40 mM NaCl, 4 mM Mg(AcO)_2_, 0.5 mM EDTA, pH 7.5) at RT. Equal amount of dialysis buffer without protein was used as control. The ATP hydrolysis reaction was terminated at 30 min by adding 200 μl reagent. After incubation for another 30 min at room temperature, the absorbance was measured at 620 nm on a plate reader (Flexstation3, Molecular Devices). The reaction velocity and ATP concentration were fitted to the Michaelis–Menten equation (*V* = *V*_max_[S]/*K*_m_ + [S]). For the ATP hydrolysis assay with DNA or RNA substrates, 30 nM or 3 μM ssDNA or ssRNA was used in the experiment as indicated. Sequences of DNA and RNA are shown below:

**Table:** 

DNA	TCCCAAGAAAGGCATCGTCAT
RNA	UUGUACUACACAAAAGUACUG
3′UTR	UGGGGAAAUCCAUGGGUCUU
5′UTR	AGUUGUUACUGUUGCUGACU
polyA	AAAAAAAAAAAAAAAAAAAA
polyU	UUUUUUUUUUUUUUUUUUUU
polyG	GGGGGGGGGGGGGGGGGGGG
polyC	CCCCCCCCCCCCCCCCCCCC

### dsRNA/dsDNA unwinding assay

The helicase activity of ZIKA NS3 and its helicase domain was monitored by dsRNA/dsDNA unwinding assay based on FRET. Two complementary DNA or RNA molecules were synthesized and labeled with Cy3 or BHQ2 respectively. The single-strand RNA or DNA oligos were solved in annealing buffer (20 mM Tris-acetate PH 7.5, 2 mM Mg(AcO)_2,_ 0.2 mM DTT, 100 mM KCl) at a concentration of 10 μM. To prepare the dsRNA/dsDNA, equal amount (molar ratio) of complementary oligos were mixed to a 500 nM final concentration and annealed by heating at 95°C for 5 min and then cooled down slowly to room temperature. For dsRNA/dsDNA unwinding, ∼200 nM purified ZIKA NS3 protein and 50 nM double-strands substrates in reaction buffer (20 mM Tris–HCl PH 7.0, 10 mM NaCl, 0.1 mg/ml BSA, 5 mM MgCl_2_, 2 mM DTT) were added into the 96-well white plate to a volume of 100 μl. After the fluorescence was stable, 5 mM ATP and 500 nM competitor (complementary to one strand) were added into wells to start the unwinding reaction. To determine the effect of NS5 on NS3 helicase activity, 100 nM NS3F or NS3H was used in the reaction, with/without the equal amount of NS5. The reaction was performed at 30°C for 30 or 60 min. The fluorescence was measured by the plate reader (Flexstation3, Molecular Devices) with settings of Ex 550 nm, Em 620 nm, cutoff 610 nm, PMT sensitivity is set as low. The initial unwinding rate is determined by a linear fit of the first 5 min of the reaction.

### Fluorescence polarization assay

Fluorescence polarization assay was performed by using 50 nM Cy3-GCGUCUUUA CGGUGCU and the indicated concentrations of protein (see Figure 3F) in the following buffer: 20 mM Tris–HCl pH 7.0, 10 mM NaCl, 0.1 mg/ml BSA, 5 mM MgCl_2_, 1 mM DTT. Fluorescence readings were taken after a 60 min incubation at 25°C using the plate reader (Flexstation3, Molecular Devices). The equipment was set as followed: Ex 530 nm, Em 570 nm, cutoff 570 nm, readings 100, PMT sensitivity high. The data were fitted to a hyperbola: ΔmP = mP_max_[NS3]/(*K*_d_ + [NS3]) (GraphPad Prism 8).

### RNA transcription, transfection and luciferase activity measurement

RNA derived from ZIKV replicon or infectious DNA clone was transcribed *in vitro* using mMESSAGEMmachine™ T7 kit (Invitrogen Cat# AM1344) according to the instruction manual. 1 μg DNA template was added into in a 20 μl reaction with an additional 2 μl of 30 mM GTP solution. The reaction mixture was incubated at 37°C for 4 h, followed by the addition of 0.5 μl DNase I (RNase free, Takara) to remove the DNA template. The RNA was extracted by RNA Fast 200 Extraction Kit (Fastagen) and stored at −80°C in aliquots.

BHK cells (in 48-well plate) were transfected with 0.5 μg viral replicon RNA using Viafect (Promega) transfection reagent. At 9 h and 36 h post-transfection, cells were lysed with 50 μl 1× Renilla luciferase lysis buffer (Promega). 10 μl lysates were mixed with 30 μl Renilla luciferase substrates to measure the luciferase signals in a luminescence microplate reader (Promega) according to the manufacturer's protocol.

### Immunofluorescence staining

Cells were fixed with 4% paraformaldehyde for 10 min and washed three times with PBS, then permeabilized with PBS containing 0.2 % Triton X-100. After blocking with 3% BSA, cells were incubated with J2 anti-dsRNA antibody at 37°C for 2 h, washed three times with PBS, followed by goat anti-mouse IgG conjugated with Alexa Fluor^®^488 at 37°C for 1 h. After three times wash with PBS, cell were stained by DAPI and mounted with prolong gold anti fade reagent. Images were captured using confocal microscope (Zeiss, Germany) at 40× magnification.

### Recombinant ZIKV infection

Vero cells were infected with culture medium containing recombinant zika virus. Forty eight hours after infection, cells were fixed and stained with anti-ZIKA envelope protein antibody.

### Real-time RT-PCR

ZIKV genomic RNA derived from infectious DNA clones were transfected into BHK-21 cells. RNA was extracted from the culture medium at 10 and 72 h by using RNA Fast 200 Kit (Fastagen) according to manufacturer's instructions. Viral load in the supernatants was measured by RT-qPCR in Bio-Rad real time thermal cycler CFX96 using one step RT-qPCR SYBR Green kit (TIANGEN). The primers for qPCR are 5′-GGTCAGCGTCCTCTCTAATAAACG-3′ and 5′-GCACCCTAGTGTC CACTTTTTCC-3′, targeting to zika NS5 gene. Reaction conditions were as followed:50°C for 30 min, 95°C for 2 min followed by 39 cycles of 94°C for 20 s, 58°C for 20 s and 68°C for 20 s. Cq is the quantification cycle as calculated by the Bio-Rad CFX Manager 2.1 based on the MIQE guidelines. Dissociation curves were performed after each PCR run to ensure that a single PCR product had been amplified. The MIQE checklist is provided in Supplementary Table S1.

### Newly transcribed RNA labeling and specific-strand RNA quantification

Newly transcribed RNA was labeled as previous described (20,21). Briefly, cells were transfected with viral RNA transcripts derived from infectious clones and then treated by s^4^U for 3 h in dark at 9 or 48 h post transfection. Then RNA was extracted by RNA Fast200 kit. s^4^U-RNA was conjugated with MTS-biotin-XX at RT for 30 min and purified by Fast200 kit to remove redundant MTS-biotin. Biotin-labled RNA was isolated by Dynabeads MyOne Streptavidin C1 magnetic beads (Life Technologies, cat. no. 65001). After three times wash, biotin-RNA was eluted and purified to remove DTT and excess salts in elute buffer. 10% input and eluted samples were reverse-transcribed by tagged-(+) strand-specific primer and quantified by real-time PCR.

### Co-Immunoprecipitation

HEK293T Cells were co-transfected with plasmids encoding ZIKV NS2ABNS3-myc and NS5-HA and then harvested at 24 h after transfection. Cell lysates were collected and Myc antibody (MBL) was added into the lysates and incubated for 2 h at 4°C, followed by adding 20 μl of protein A/G beads to the protein solution mixture and incubate for 2 h at 4°C with gentle rotation. The mixture was centrifuged at 12 000 rpm for 2 min, and supernatant was discarded. Protein A/G beads were washed three times with wash buffer (20 mM Tris–HCl PH 8.0, 0.1 mM EDTA, 300 mM NaCl). Then the beads were resuspended in 1× protein loading buffer, and boiled for 10 min. The supernatant was analyzed by western blot. For co-immunoprecipitation experiment using purified protein, equal amount (molar ratio) of purified NS3 and NS5 proteins were incubated for 2 h at 4°C and then same co-immunoprecipitation procedure was carried out.

### Statistical analysis

The error bars in the figures represent the standard error of mean of three repeats or duplicates as indicated. The statistical significance of differences between groups was evaluated by the two-tailed unpaired Student's *t*-test. *P-value < 0.05 was considered statistically significant. **P-value < 0.01 was very significant and ***P-value < 0.001 was extremely significant.

## RESULTS

### ZIKV NS3 has intrinsic ATPase activity

**Figure 1.**
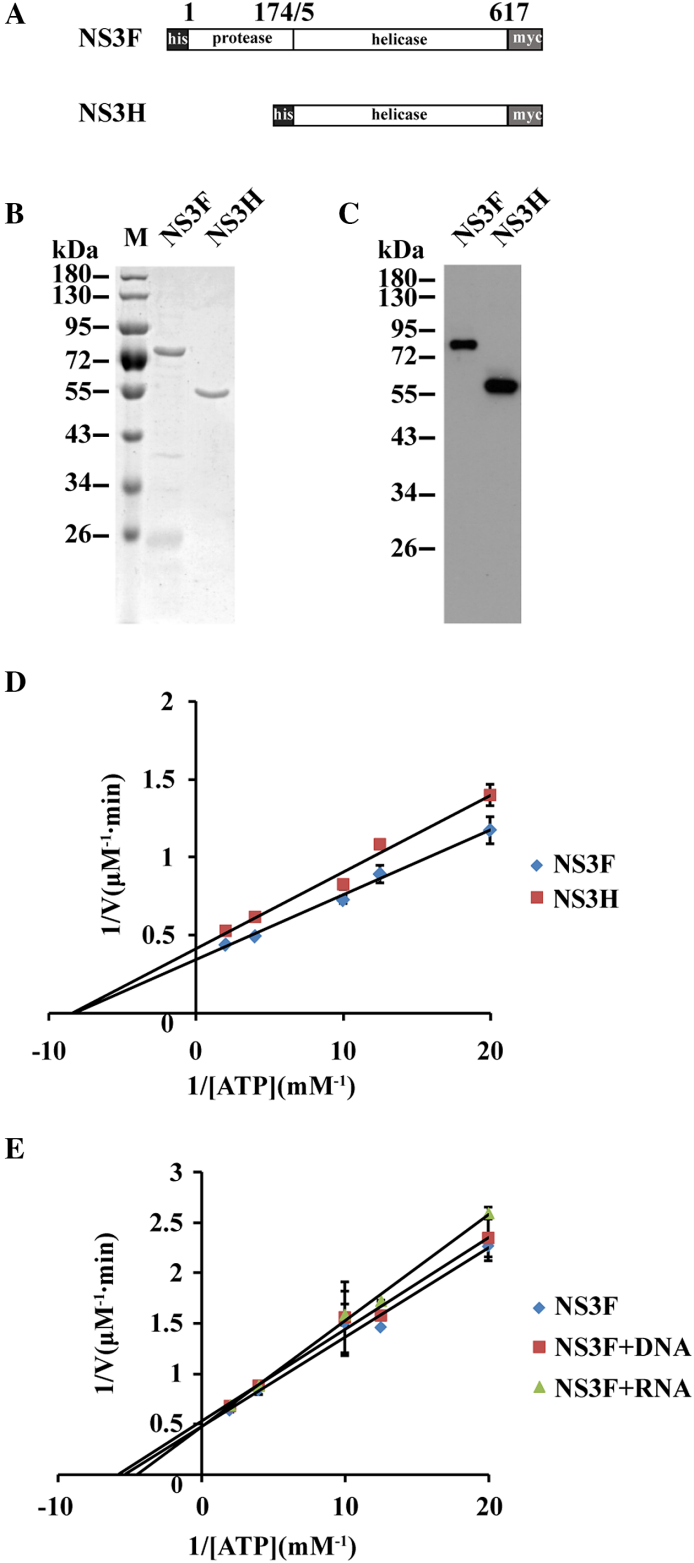
Purification and ATPase activity analysis of ZIKV NS3F (NS3 full) and NS3H (NS3 helicase). (**A**) Schematic representation of recombinant ZIKV NS3F (1–617 residues) and NS3H proteins (175–617 residues). (**B**) SDS-PAGE analysis of the purified NS3F and NS3H proteins. Proteins were stained with Coomassie Blue. (**C**) Western blot analysis of the purified NS3F and NS3H proteins with an anti-myc antibody. (**D**) The ATPase activity of the purified NS3F and NS3H proteins. The ATP hydrolysis assay was carried out with 30 nM enzyme in the presence of the indicated concentrations of ATP for 30 min at room temperature. The double reciprocal plot was fitted according to the Michaelis–Menten equation. Error bars represent the standard error of duplicate measurements. (**E**) The ATPase activity of ZIKV NS3F in the absence or presence of ssDNA or ssRNA. 30 nM ssDNA or ssRNA was used in the ATP hydrolysis assay. Error bars represent the standard error of duplicate measurements.

ZIKV NS3 has two functional domains, the N-terminal protease domain (residues 1–174) and the C-terminal helicase domain (NS3H, residues 175–617, Figure 1A). To characterize ZIKV NS3 helicase activity, we expressed and purified full-length NS3 (NS3F, residues 2–617) and its helicase domain (NS3H, residues 175–617) from *E. coli*. Coomassie Blue staining and Western blotting showed that both proteins had correct molecular weights, consistent with the theoretical calculation (Figure 1B and C). Helicases usually hydrolyze ATP to provide energy for unwinding DNA or RNA duplexes (22–24). We first examined the ATPase activity of ZIKV NS3 using a colorimetric assay with the Malachite Green reagent, which can detect free inorganic phosphate released in the ATP hydrolysis reaction. The data were presented as a double reciprocal plot that was fitted according to the Michaelis–Menten equation. As shown in Figure 1D, the purified full-length NS3 and its helicase domain had similar ATP hydrolase activity (NS3F: *V*_max_ = 2.76 μmol /l·min and *K*_m_ = 0.11 mmol/l, NS3H: *V*_max_ = 2.37 μmol /l·min and *K*_m_ = 0.12 mmol/l). The N-terminal protease domain appears to have a very weak effect on ATPase activity. Adding DNA or RNA substrate (30 nM) into ATP hydrolysis reaction did not apparently affect NS3 ATPase activity (Figure 1E and Supplementary Figure S1). Thus, similar to other DExH family helicases, ZIKV NS3 has intrinsic ATPase activity without RNA/DNA binding (23,25,26).

### ZIKV NS3 selectively unwinds dsDNA/RNA with 3′overhangs

**Figure 2.**
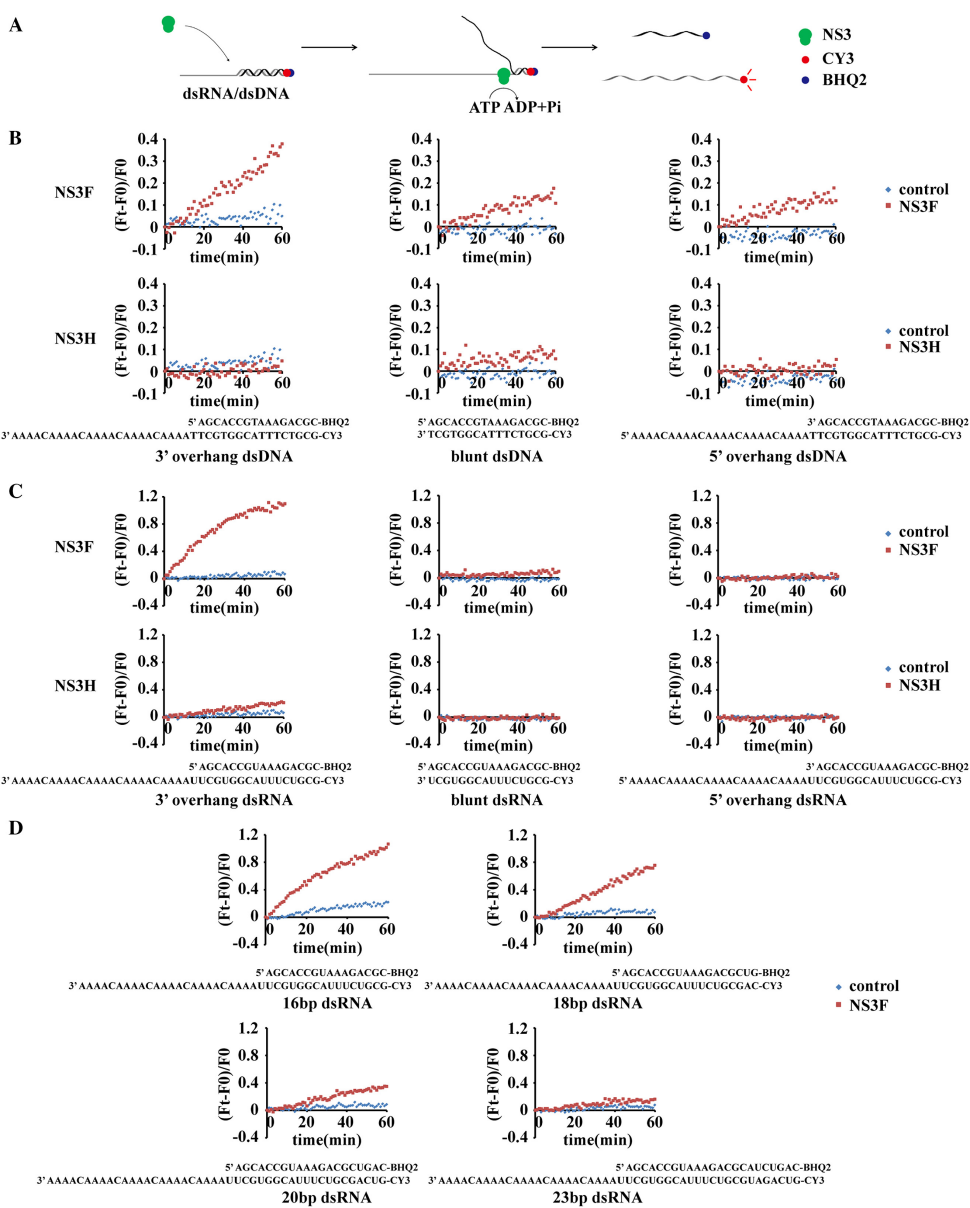
FRET-based dsDNA/RNA unwinding by NS3F and NS3H proteins. (**A**) Schematic illustration of the fluorescence resonance energy transfer (FRET)-based duplex unwinding assay. The two complementary nucleic acid strands are labeled separately with Cy3 and Black Hole Quencher-2 (BHQ2). When the complementary strands are annealed into double strands, the fluorescence emitted by Cy3 is quenched by BHQ2. With the opening of duplexes by the NS3 protein with ATP, the quenching of Cy3 by BHQ2 is relieved; thus, Cy3 fluorescence increases accordingly. (B and C) The real-time unwinding kinetics of ZIKV NS3F and NS3H on 16 bp dsDNA (**B**) or 16 bp dsRNA (**C**) with a 3′ overhang, blunt ends or a 5′ overhang. Reactions without enzyme were used as controls. The fluorescent signal was measured every 1 min. The unwinding activity was defined as (*F*_t_ – *F*_0_)/*F*_0_, where *F*_t_ is the fluorescence of the sample at a given time, and *F*_0_ is the initial fluorescence of the sample. The sequence information of dsDNA/RNA is shown at the bottom of each assay. (**D**) The real-time unwinding kinetics of NS3F on dsRNA with different lengths (16, 18, 20 and 23 bp). 200 nM enzyme and 50 nM duplexes were used in the unwinding reactions mentioned above.

Next, we tested whether ZIKV NS3 has helicase activity *in vitro*. An unwinding assay based on the FRET effect was set up to monitor the separation of the double-stranded molecule by ZIKV NS3 in real time (Figure 2A) (27). The two complementary nucleic acid strands were labeled with Cy3 or Black Hole Quencher-2 (BHQ2). When the complementary strands were annealed into double strands, the fluorescence emitted by Cy3 was mostly absorbed by BHQ2, resulting in low Cy3 fluorescent signals. With the opening of the duplexes by helicase, the quenching of Cy3 by BHQ2 is relieved; thus, Cy3 fluorescence increases accordingly (Figure 2A). The FRET-based unwinding assay has evident advantages in its temporal resolution and quantification.

To understand the selectivity and polarity of ZIKV NS3 during substrate unwinding, three pairs of complementary RNA/DNA strands were synthesized and annealed to produce dsRNA/DNA with a 3′ overhang, a blunt end or a 5′ overhang (Figure 2B and C). We found that ZIKV NS3 unwound both dsRNA and dsDNA *in vitro*, even though only dsRNA is its *bona fide* substrate during viral replication. In addition, ZIKV NS3 preferred to unwind dsRNA and dsDNA that had a 3′ overhang (Figure 2B and C), with weak unwinding activity for substrates with blunt ends or a 5′ overhang. This finding indicated the 3′ to 5′ directionality of the enzyme, suggesting that the 3′overhang serves as the loading strand. Interestingly, although similar ATPase activity was shown between NS3H and NS3F, NS3H displayed much weaker helicase activity than NS3F at the same concentration (Figure 2B and C). Active DNA/RNA unwinding could be observed only if a high concentration of NS3H proteins (3μM) was used in the assay (Supplementary Figure S2). This finding suggests that the NS3 protease domain could be an intramolecular cofactor that helps the NS3 helicase domain to open the duplex, probably through either stabilizing the helicase conformation or promoting binding between helicase and the substrate (28).

To avoid ATP-independent dsRNA/DNA unwinding under high helicase-to-substrate ratio condition (29), we used 4:1 helicase-to-substrate ratio in the unwinding assay, which is much lower than that used in earlier studies (30–33). At this ratio, no ATP-independent dsRNA unwinding or protein induced fluorescence enhancement (PIFE) was observed (Supplementary Figure S3A). This also resulted in a relatively low unwound fraction (about 7%). When helicase-to-substrate ratio was increased to 100:1, the unwinding fraction can be elevated to about 40% (Supplementary Figure S3B).

We further explored whether the length of substrate affected unwinding reaction of ZIKV NS3. The data showed that with increasing length of dsRNA, the unwinding activity of NS3 drops dramatically (Figure 2D). NS3 almost lost its ability to separate the 23 bp RNA duplex. Our unwinding assay is an ‘all-or-none’ reaction in consideration of its ‘end-point’ signal detection, while partially separated duplexes do not cause fluorescence changes. Therefore, it is possible that NS3 partially opened the longer RNA duplex but failed to finish the translocation throughout the full-length RNA strand, probably due to insufficient processivity. This is consistent with previous reports that hepatitis C virus (HCV) NS3 would drop or slip backward from the duplex at 18 bp, where it encountered an energy barrier (34,35).

### ZIKV NS3 helicase activity relies on its ATPase activity and substrate binding

**Figure 3.**
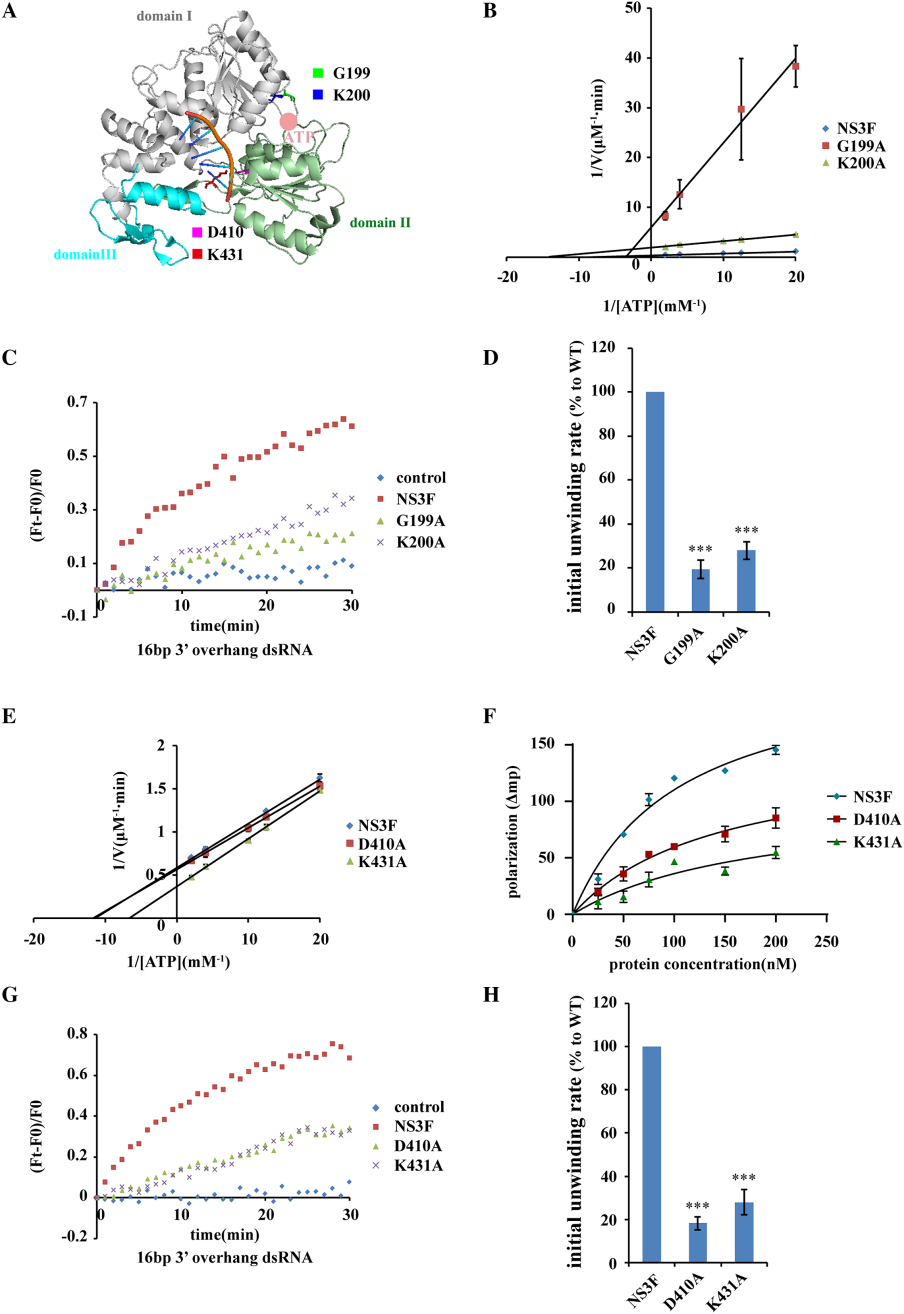
The helicase activity of NS3 depends on ATP hydrolysis and RNA binding. (**A**) The residues identified as important for NS3 helicase activity reside in the ATP and RNA binding sites (PDB: 5GJB (36)). The residues are shown as sticks in the indicated colors. Single-strand RNA is shown in orange. (**B**) The ATPase activity of WT and mutated NS3 (G199A and K200A). The ATP hydrolysis assay was carried out with 30 nM enzyme in the presence of the indicated concentrations of ATP. (**C**) The dsRNA unwinding kinetics of WT and mutated NS3 (G199A and K200A). 16bp dsRNA with 3′overhang was used as substrate. 200 nM enzyme and 50 nM duplexes were used in the unwinding reactions. (**D**) Quantification of the initial dsRNA unwinding rate of WT or mutated NS3 in (C). The initial unwinding rate is calculated by a linear fit of the first 5 min of the reaction, and the initial unwinding rate of NS3F is defined as 100%. (**E**) The ATPase activity of WT and mutated NS3 (D410A and K431A). (**F**) Fluorescence polarization assay to detect the binding of ssRNA and WT or mutated NS3 (D410A and K431A). The assay was carried out with 50 nM ssRNA in the presence of the indicated concentrations of enzyme. The data were fitted to a hyperbola, Kd_NS3F_ = 99 ± 7 nM, Kd_D410A_ = 140 ± 6 nM, Kd_K431A_ = 160 ± 20 nM. (**G**) The dsRNA unwinding kinetics of WT and mutated NS3 (D410A and K431A). 16 bp dsRNA with 3′ overhang was used as substrate. 200 nM enzyme and 50 nM duplexes were used in the unwinding reactions. (**H**) Quantification of the initial dsRNA unwinding rate of WT or mutated NS3 in (G). The initial unwinding rate is calculated by a linear fit of the first 5 min of the reaction, and the initial unwinding rate of NS3F is defined as 100%. Error bars represent the standard error of duplicate measurements (B and E) or triplicate measurements (D, F and H) (***P < 0.001).

Does ZIKV NS3 helicase activity rely on ATP hydrolysis? To answer this question, we constructed NS3 ATPase inactive mutants and examined their helicase activity. Sequence alignment of NS3 proteins from flavivirus family members (Supplementary Figure S4) demonstrates that residues G199 and K200 are highly conserved, and reside in the NS3 ATP binding motif (Figure 3A) (7,17,36–38). ATP hydrolysis assays showed that both G199A and K200A mutations significantly impaired NS3 ATPase activity (Figure 3B). Accordingly, when these two mutants were applied in the dsRNA unwinding assay, they displayed attenuated RNA helicase activity with slower unwinding rate (Figure 3C and D), indicating that NS3 helicase activity relies on ATP hydrolysis.

Additionally, we introduced mutations into the RNA binding region of NS3 to interrupt its recognition of RNA. Structural studies showed that single strand RNA binds to a positive charged tunnel surrounded by subdomain I, II and III of the NS3 helicase domain (36,37), where residues D410 and K431 of NS3 interacted with each other and formed hydrogen bonds with RNA (Figure 3A). Therefore, we assumed that these two residues are important for the interaction between NS3 and RNA. We mutated D410 and K431 to alanine and test the enzymatic activity of the mutants. Similar (D410A) or slightly increased ATPase activity (K431A) was detected compared to wild type (WT) NS3 (Figure 3E), implying that these mutations did not inhibit NS3 ATPase activity, which is reasonable because these two residues are far from the ATP binding site. However, these two mutations impaired NS3 association with RNA (Figure 3F). Consistently, in the dsRNA unwinding assay, both mutants showed decreased helicase activity compared to that of WT NS3 (Figure 3G and H). Clearly, disrupting the interaction between NS3 and the RNA substrate also affects its helicase activity. Conclusively, ZIKV NS3 unwinds the RNA duplex in a manner dependent on ATP hydrolysis and RNA binding.

### ZIKV NS3 helicase activity is essential for viral replication

**Figure 4.**
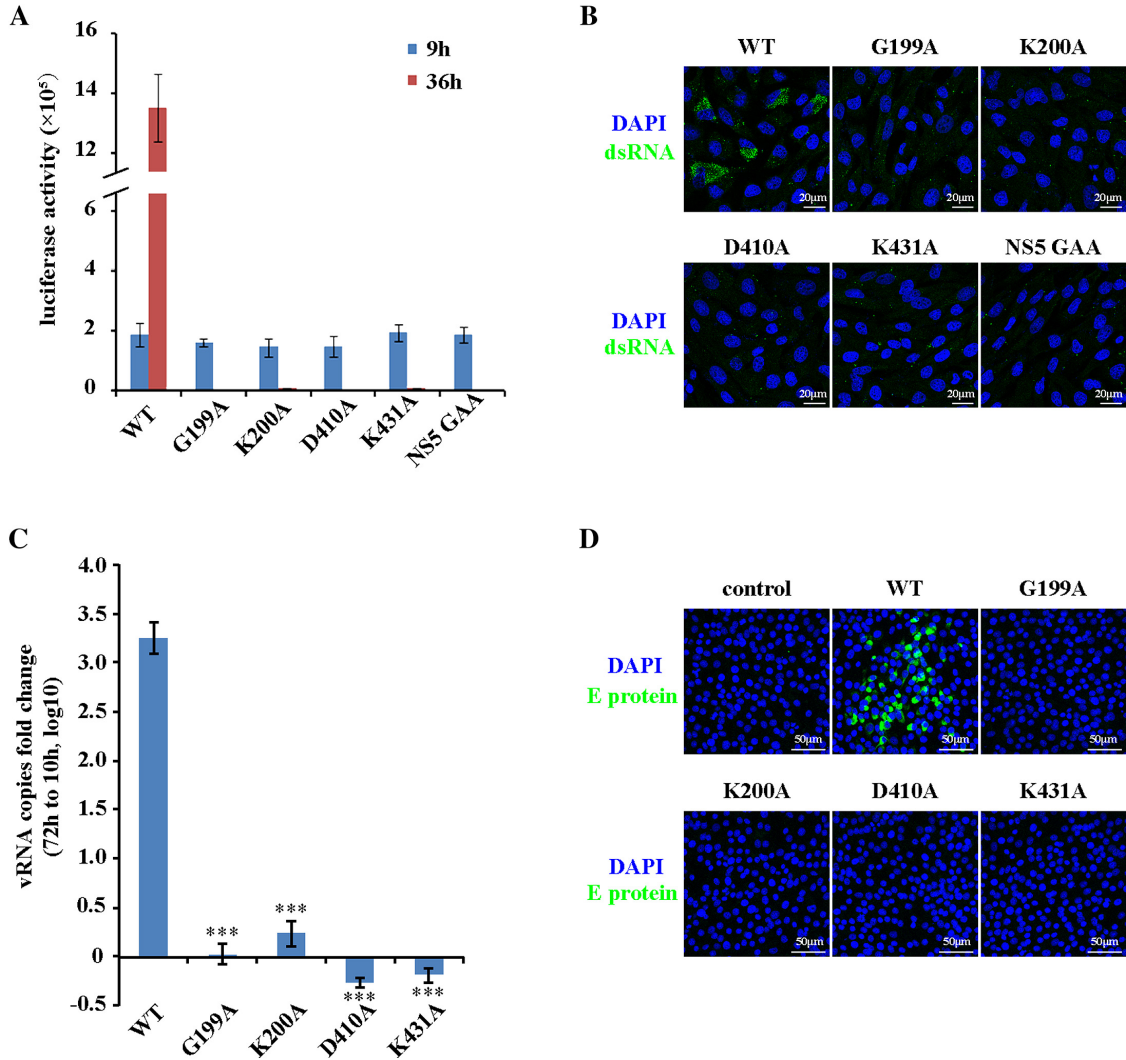
ZIKV NS3 is essential for viral replication. (**A**) Luciferase assay based on ZIKV replicon. BHK-21 cells were transfected with WT or NS3-mutated ZIKV replicon RNA, and luciferase activity was measured at 9 h and 36 h post transfection. (**B**) Immunostaining to detect viral dsRNA. BHK-21 cells were transfected with the WT or NS3 mutated ZIKV replicon, and viral dsRNA was stained with the J2 antibody. (**C**) Recombinant ZIKV released into the culture medium was quantified by RT-qPCR after transfection of WT or NS3 mutated infectious ZIKV genomic RNA into BHK-21 cells (***P < 0.001). (**D**) Infection assay with recombinant ZIKV. BHK-21 cells were transfected with WT or NS3-mutated infectious ZIKV genomic RNA, and recombinant ZIKV was collected to infect Vero cells again. Infected Vero cells were stained with an antibody specific for the ZIKV E protein. Error bars represent the standard error of triplicate measurements (A and C).

We have shown that ZIKV NS3 has ATPase and RNA helicase activity and characterized its enzymatic properties *in vitro*. During ZIKV replication in the cell, NS3 unwinds the dsRNA intermediate to ensure that RNA synthesis is subsequently carried out by NS5. We hypothesized that the same unwinding mechanism revealed by our *in vitro* assay would apply to NS3 during ZIKV replication *in vivo*. To test this idea, we examined viral replication using a luciferase reporter assay based on the ZIKV replicon (39). The ZIKV replicon can mimic viral replication in the cell, and contains the genomic RNA sequence except for the viral structural proteins, which are replaced by the Renilla Luciferase reporter. Thus, the luciferase activity can be detected to monitor viral replication in the cell. In the assay, an immediate luciferase activity (9 h post transfection) comes from the protein translation of transfected replicon RNA. Only if the replicon successfully replicates in cells, a robust luciferase activity (36 h post transfection) could be detected. We introduced NS3 mutations (G199A, K200A, D410A and K431A) that affect NS3 helicase activity *in vitro* into the ZIKV replicon and detected their luciferase activity. At 9 h post transfection, both WT and mutated replicons showed similar luciferase activity, suggesting similar RNA transfection efficiency and protein translation level. The WT ZIKV replicon showed robust luciferase activity at 36 h post transfection, indicating successful replication in BHK-21 cells (Figure 4A and Supplementary Figure S7). A replication-incompetent mutant, NS5 GAA (RNA polymerase inactive mutation, 664–666 residues, GDD to GAA) was used as a negative control and showed very little luciferase signal at 36 h post transfection. As shown in Figure 4A, all NS3-mutated replicons displayed very low luciferase activity at 36 h, implying little to no viral replication.

To confirm that NS3 mutations lead to the failure of viral RNA amplification, we stained the cells with the J2 antibody, which specifically recognized dsRNA, after transfection with the WT or mutated ZIKV replicon. Consistent with the results from the luciferase assay, dsRNA was detected in BHK cells transfected with the WT ZIKV replicon (Figure 4B). However, few fluorescent signals were observed in the groups transfected with either NS3-mutated replicons or NS5-mutated replicon (Figure 4B), affirming that the NS3 mutations blocked viral replication at the step of RNA synthesis.

The crucial role of NS3 in ZIKV production was further evaluated via preparation of recombinant ZIKV (rZIKV) (40). An infectious ZIKV cDNA clone was used as a template for viral genomic RNA transcription *in vitro* (Supplementary Figure S7). Viral RNA was then transfected into BHK cells to produce rZIKV, and rZIKV released into culture medium was collected and quantified by qPCR. WT infectious cDNA clone derived viral RNA successfully produced rZIKV, which could infect Vero cells again (Figure 4C and D). All NS3 mutations (G199A, K200A, D410A and K431A) failed to produce rZIKV (Figure 4C and D). We labeled and purified newly transcribed RNA containing 4-thiouridine (s^4^U) and detected viral RNA by RT-qPCR at 48 h post transfection, and found that NS3 mutations severely inhibited viral RNA synthesis in cells (Supplementary Figure S8) (20,21,41). Collectively, these results confirmed that ZIKV NS3 helicase activity is essential for viral replication, which requires ATP hydrolysis and substrate binding.

### ZIKV NS3 helicase activity is stimulated by NS5

**Figure 5.**
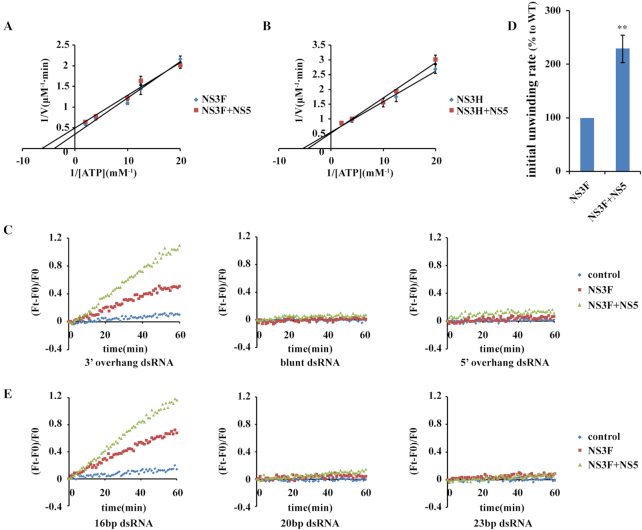
NS5 stimulates NS3 helicase activity. (A and B) The ATPase activity of NS3F (**A**) and NS3H (**B**) with or without NS5. The ATP hydrolysis assay by 30 nM NS3F or NS3H was carried out with/without 30 nM NS5 in the presence of the indicated concentrations of ATP. Error bars represent the standard error of duplicate measurements. (**C**) Duplex unwinding assay of NS3F with/without NS5 on 16 bp dsRNA with a 3′ overhang, blunt ends or a 5′ overhang. (**D**) Quantification of the initial dsRNA unwinding rate of NS3 with/without NS5 in the left panel of (C). The initial unwinding rate is calculated by a linear fit of the first 5 min of the reaction, and the initial unwinding rate of NS3F is defined as 100%. Error bars represent the standard error of triplicate measurements (**P < 0.01). (E) Duplex unwinding assay of NS3F with/without NS5 on dsRNA of different lengths with 3′ overhangs. The unwinding reactions were performed with 100 nM NS3F and 100 nM NS5.

Flaviviral replication is accomplished by multicomponent complexes, in which two enzymatic proteins, NS3 and NS5 play important roles (42). During viral RNA replication, dsRNA unwinding by NS3 is essential for almost all NS5-dominated RNA synthesis, except for the first round. It is likely that dsRNA unwinding and subsequent RNA synthesis are coupled to each other for efficient viral replication (31,43,44). Thus, the hypothesis that NS5 could act as a cofactor to facilitate NS3 function is reasonable. To test this, we purified ZIKV NS5 proteins and examined whether NS5 affects NS3 helicase activity. NS5 did not influence the ATPase activity of either the full-length NS3 or the NS3 helicase domain (Figure 5A and B). We used half amount of NS3 (100 nM) in the unwinding assay to examine the effect of NS5 and we found that NS5 specifically stimulated NS3 helicase activity during the opening of dsRNA with a 3′ overhang, but not dsRNA with blunt ends or a 5′ overhang (Figure 5C and D). Similarly, NS5 also specifically stimulated NS3 to unwind dsDNA with a 3′overhang (Supplementary Figure S5). Additionally, NS5 cannot increase Cy3 signal by itself ruling out the PIFE effect derived from NS5 proteins (Supplementary Figure S3A). Interestingly, NS5 selectively facilitated the unwinding of dsDNA, but not dsRNA by NS3H (Supplementary Figure S6), suggesting specific substrate recognition.

Increasing the kinetics of RNA unwinding means that more duplexes are opened per unit time, which could occur via two possibilities. The first possibility is that NS3 walks ‘faster’ on the RNA strand with NS5 assistance. The other one is that NS5 improves the processivity of NS3, namely, more NS3 successfully walks through the full-length RNA duplex rather than dropping off the RNA duplex in the middle. We then tested whether NS5 could promote NS3 helicase activity to unwind longer dsRNA and found that no obvious improvement was observed when 20 or 23 bp dsRNA probe was used as a substrate (Figure 5E), ruling out the latter possibility. Together, these data suggest that NS5 stimulates NS3 helicase activity through increasing its unwinding velocity rather than improving its processivity on the nucleic acid strand.

### The ZIKV NS3-NS5 interaction is crucial for dsRNA unwinding during viral replication

**Figure 6.**
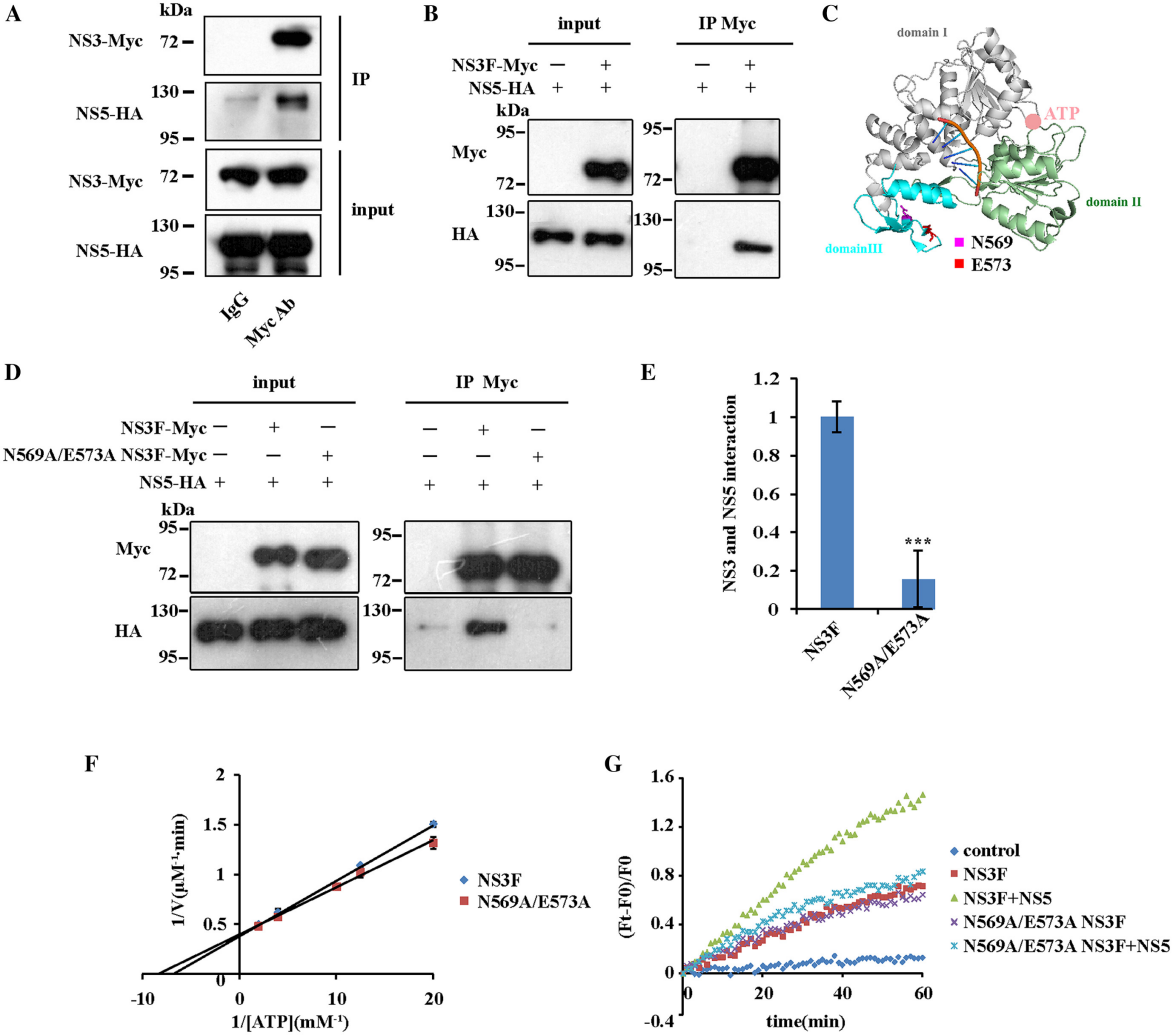
The facilitation of NS3 helicase activity by NS5 depends on the NS3-NS5 interaction. (**A**) ZIKV NS3 interacts with NS5 in cells. NS3-Myc and NS5-HA were co-expressed in HEK293T cells and their interaction was examined by Co-Immunoprecipitation. Gel blots were probed with anti-Myc or anti-HA antibody. (**B**) ZIKV NS3 directly interacts with NS5. Co-immunoprecipitation was carried out with the purified NS3 and NS5 proteins and then analyzed by Western blotting. (**C**) Residues Asn-569 and Glu-573 lie far away from the ATP and RNA binding sites of NS3 (PDB: 5GJB (36)). The RNA is shown in orange, and ATP is shown as pink dot. (**D**) The N569A/E573A mutation interrupts the NS3-NS5 interaction. Co-immunoprecipitation was carried out with NS5 and WT or mutated NS3 proteins and then analyzed by western blotting. (**E**) Quantification of the interaction between NS5 and WT or mutated NS3 in (D) (***P < 0.001). Error bars represent the standard error of triplicate measurements. (**F**) The N569A/E573A mutation does not affect NS3 ATPase activity. 30 nM enzyme was used to hydrolyze ATP. Error bars represent the standard error of duplicate measurements. (**G**) dsRNA unwinding assay of WT or N569A/E573A mutated NS3 with/without NS5. 16 bp dsRNA with 3′ overhang was used as substrate. The reactions were carried out with 100 nM NS3F (or mutants) and 100 nM NS5.

Previous studies reported that the interaction between NS3 and NS5 is important for viral replication in HCV and DENV (45–47). Does ZIKV NS3 also interact with NS5? We performed co-immunoprecipitation and found that they interacted with each other in cells (Figure 6A). In addition, co-immunoprecipitation using purified ZIKV NS3 and NS5 proteins indicated a direct interaction between them (Figure 6B). Then we tried to determine whether NS5-simulated NS3 helicase activity depends on their interaction. To this end, we need to introduce mutations into NS3 protein that could disrupt the NS3-NS5 interaction without affecting the enzymatic function of NS3. The C-terminal region of the DENV NS3 protein has been reported to be important for NS5 binding, among which the peptide containing residues 566–585 is far from the ATP and RNA binding region according to the crystal structure (Figure 6C) (48). Therefore, a mutation in this peptide is likely to disrupt the NS3-NS5 interaction, while maintaining NS3 enzymatic function. Two conserved residues, N569 and E573, were mutated to alanine (Supplementary Figure S4), and this mutant's interaction with NS5 and its enzymatic activity was tested. The N569A/E573A mutation significantly interrupted the NS3-NS5 interaction, as indicated by co-immunoprecipitation (Figure 6D and E). As we predicted, the N569A/E573A mutation affected neither ATP hydrolysis nor dsRNA unwinding of NS3 (Figure 6F and G). However, NS5 could no longer facilitate helicase activity of mutated NS3 in the RNA unwinding assay as observed for WT NS3 (Figure 6G), indicating that the promotion of NS3 helicase activity by NS5 relies on the interaction of the two proteins.

**Figure 7.**
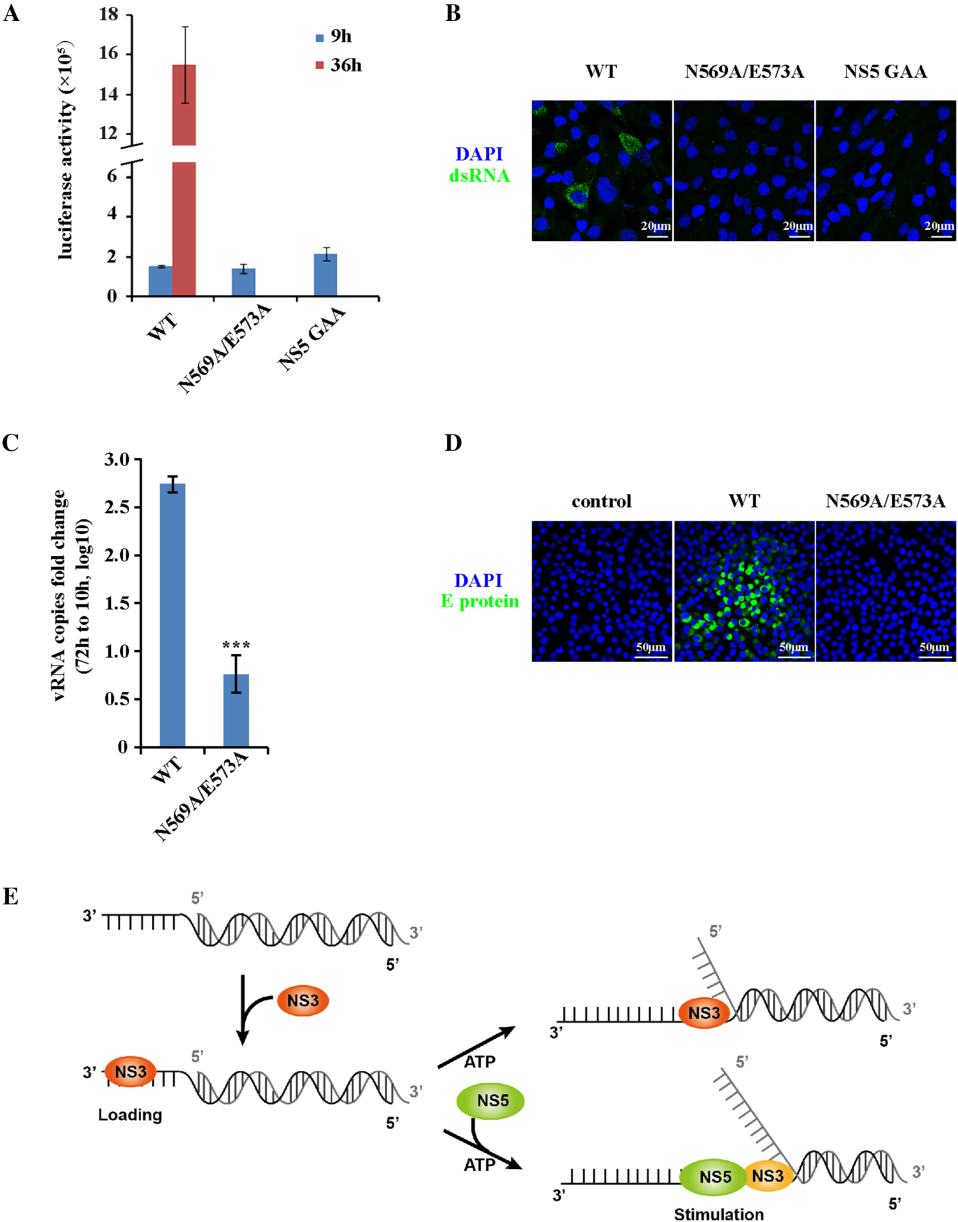
The NS3-NS5 interaction is crucial for viral replication. (**A**) Luciferase assay of the ZIKV replicon. BHK-21 cells were transfected with WT or NS3 N569A/E573A-mutated ZIKV replicon RNA, and luciferase activity was measured at 9 h and 36 h post transfection. Error bars represent the standard error of triplicate measurements. (**B**) Immunostaining to detect viral dsRNA after transfection of the WT or NS3-mutated ZIKV replicon in BHK-21 cells. (**C**) Quantification of recombinant ZIKV released into the culture medium after transfection of infectious WT or NS3-mutated ZIKV genomic RNA into BHK-21 cells by RT-qPCR (***P < 0.001). Error bars represent the standard error of triplicate measurements. (**D**) Infection assay with recombinant ZIKV. BHK-21 cells were transfected with WT or mutated infectious ZIKV genomic RNA, and recombinant ZIKV was collected to infect Vero cells again. Infected Vero cells were stained with an antibody specific for the ZIKV E protein. (**E**) ZIKV NS3 is a canonical RNA helicase stimulated by NS5 RNA polymerase. NS3 first loads onto the single stranded region of viral dsRNA with 3′overhang. Depending on ATP hydrolysis, NS3 unwinds dsRNA in the 3′ to 5′ direction. ZIKV NS5, the RNA dependent RNA polymerase, can stimulate NS3 helicase activity. The tight coupling of dsRNA unwinding by NS3 and RNA synthesis by NS5 ensures the high efficiency of viral replication.

The connection of ZIKV NS3 helicase activity to NS5 *in vitro* suggests that the cooperation between them might be important for viral replication. Thus, disrupting their interaction may lead to defects in viral replication. We performed a ZIKV replicon luciferase assay with the NS3 N569A/E573A mutation and found that the mutation blocked viral replication (Figure 7A and Supplementary Figure S7). Immunostaining for dsRNA verified that viral RNA synthesis was blocked when the NS3 N569/E573 residues were mutated (Figure 7B). Moreover, the NS3 N569A/E573Amutation significantly decreased recombinant ZIKV production because of viral RNA synthesis deficiency (Figure 7C, D and Supplementary Figure S8). Of course, we cannot rule out the possibility that NS3 may also facilitate NS5 polymerase activity during viral RNA synthesis (49). Disrupting the interaction of these proteins might also affect the RNA synthesis step. Thus, although individual enzymatic functions of NS3 and NS5 were remained intact, uncoupling dsRNA unwinding and RNA synthesis by interrupting the NS3-NS5 interaction still results in serious viral replication defects. Combined with our results *in vitro*, we suggest that NS5 is a key cofactor of NS3, whose facilitation of NS3 helicase activity is crucial for ZIKV replication.

## DISCUSSION

ZIKV NS3 belongs to the superfamily 2 (SF2) DExH group helicase, like its homologues in other flavivirus family members (23). Here, we systematically characterized the enzymatic activity of the ZIKV NS3 protein, which plays an essential role in ZIKV replication with the assistance of NS5. We found that NS3 possessed intrinsic ATPase activity, a feature also observed for HCV NS3 protein (26,50). Earlier reports showed that oligonucleotides such as poly (A) and poly (U) stimulated flaviviral NS3 ATPase activity (31,50). We did not observe apparent stimulation of NS3 ATPase activity by oligonucleotides probably because the oligonucleotides concentration we used (30 nM or 3 μM) is much lower than others (several hundred μM). We cannot rule out the possibility that oligonucleotides would stimulate NS3 ATPase activity under some conditions.

Our FRET assay directly provided real-time dsRNA/dsDNA unwinding kinetics for ZIKV NS3. We showed that ZIKV NS3 unwound both DNA and RNA duplexes in vitro without specific selectivity. We mainly focus on the unwinding of dsRNA by NS3 because the dsRNA intermediate is the bona fide substrate during flaviviral replication. Our data clearly supported the idea that ZIKV NS3 is a canonical RNA helicase with specific polarity, which requires a 3′ flanked single-strand as a loading strand and unwinds dsRNA or dsDNA in the 3′ to 5′ direction (51). On the issue of whether the N-terminal segregated protease domain affects ATPase and helicase activity, earlier studies on NS3 from different Flaviviridae members provided controversial conclusions (13,32,52,53). One study compared ATPase and helicase activity of NS2B-NS3 and NS3 helicase domain from Murray Valley encephalitis virus, while no apparent difference between them was observed (13). In another report, DENV NS3 protease domain seemed to inhibit NS3 ATPase activity (32). In contrast, Luo et al showed that DENV full length NS2B18NS3 had higher ATPase and helicase activity than NS3 helicase domain, and the linker between protease and helicase domain displayed functional significance (53). Similarly, protease domain of HCV NS3 stimulated its helicase activity (52). We found that although the ZIKV NS3 protease domain does not change NS3 ATPase activity, it is necessary for optimal helicase activity.

We also identified several key residues for the helicase activity of ZIKV NS3. These residues are located in either the ATP binding site or RNA binding region, whose mutations impair NS3 helicase activity *in vitro* and severely interrupt viral replication in the cell. Evidently, the unwinding of dsRNA (helicase activity) by NS3 requires energy from ATP hydrolysis (ATPase activity), although its ATPase activity is independent of RNA binding. Notably, some NS3 mutants displayed even more serious defects in viral replication than the relative mild impacts on NS3 helicase activity that were observed in the RNA unwinding assay. This discrepancy could be because unwinding a much longer viral dsRNA intermediate (∼11 kb) is more difficult than unwinding the 16bp dsRNA probe used in our unwinding assay (54). Therefore, even a mild compromise in NS3 helicase activity could lead to the abortion of viral replication, suggesting that optimal NS3 activity is necessary for successful viral replication under stringent cellular conditions.

Interestingly, it turned out that ZIKV NS3 cannot efficiently open dsRNA longer than 18 bp, implying that it has limited processivity in duplex unwinding. The poor processivity of ZIKV NS3 makes it difficult to imagine that a single helicase alone could unwind the 11 kb long viral dsRNA intermediate. In this case, persistent unwinding of viral dsRNA would require either the cooperation of multiple monomeric/oligomeric NS3 molecules or cofactors that improve the activity of NS3 on RNA strand (55–58). Considering that dsRNA unwinding and RNA synthesis are closely connected steps in flavivirus replication, we examined whether the ZIKV polymerase, the NS5 protein, is a cofactor of NS3. We found that NS5 interacts with NS3 and promotes NS3 helicase activity, but not ATPase activity. Kinetic analysis indicated that NS5 could increase NS3 unwinding velocity without improving its processivity on the RNA duplex. Increasing evidence argues that helicases are often regulated by a variety of cofactors in multiple processes (33,59). In particular, as DNA/RNA amplification is usually coupled to duplex unwinding, the facilitation of helicase activity by polymerase is generally observed, between DNA polymerases and helicases in eukaryotes, bacteria and viruses (31,60–62). Combining our results and other studies, we propose a ZIKV dsRNA unwinding model (Figure 7E). ZIKV NS3 is a canonical helicase that loads on the 3′ overhang of dsRNA, and then unwinds viral dsRNA in the 3′ to 5′ direction in an ATP-dependent manner. ZIKV NS5 stimulates unwinding activity of NS3 probably by facilitating the catalytically active conformation transition of NS3, or destabilizing the RNA duplex. The NS3-NS5 interaction may couple the unwinding step to the synthesis step. Thus, NS5 facilitates NS3 unwinding the viral dsRNA and synthesizes new RNA strand, which could be an optimized strategy for viral replication.

In summary, our work characterized the helicase activity of ZIKV NS3 and revealed its essential role in ZIKV replication. NS3 helicase activity can be stimulated by NS5, providing novel insight into ZIKV replication. Single molecular studies of flaviviral NS3 translocation on the RNA strand may offer more in-depth understandings in the future.

## Supplementary Material

gkz650_Supplemental_Files
