# Supplementary material for: Zika virus NS3 is a canonical RNA helicase stimulated by NS5 RNA polymerase
Source: Nucleic Acids Res. 2019 Jul 30;47(16):8693–707. doi: 10.1093/nar/gkz650 (PMC6895266; doi:10.1093/nar/gkz650)
Supplement: gkz650_Supplemental_Files [file gkz650_supplemental_files.zip › 190709 supplementary data clean.docx]

SUPPLEMENTARY DATA­


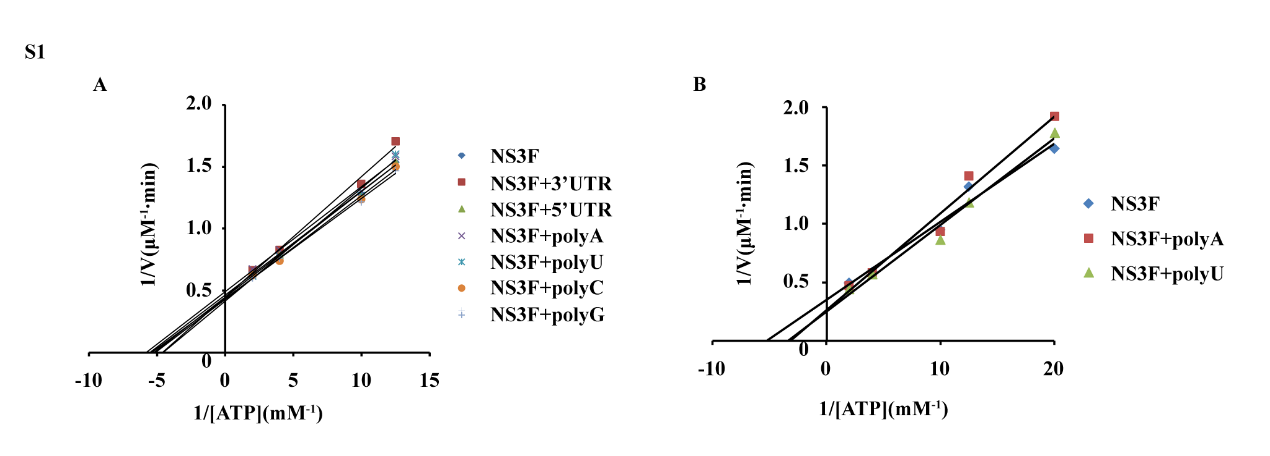


**Figure S1.** The ATPase activity of ZIKV NS3F in the absence or presence of various ssRNAs. The ATP hydrolysis assay was carried out with 30 nM NS3F in the presence of the indicated concentrations of ATP. (A) 30 nM or (B) 3 μM ssRNA as indicated was used in the ATP hydrolysis assay.


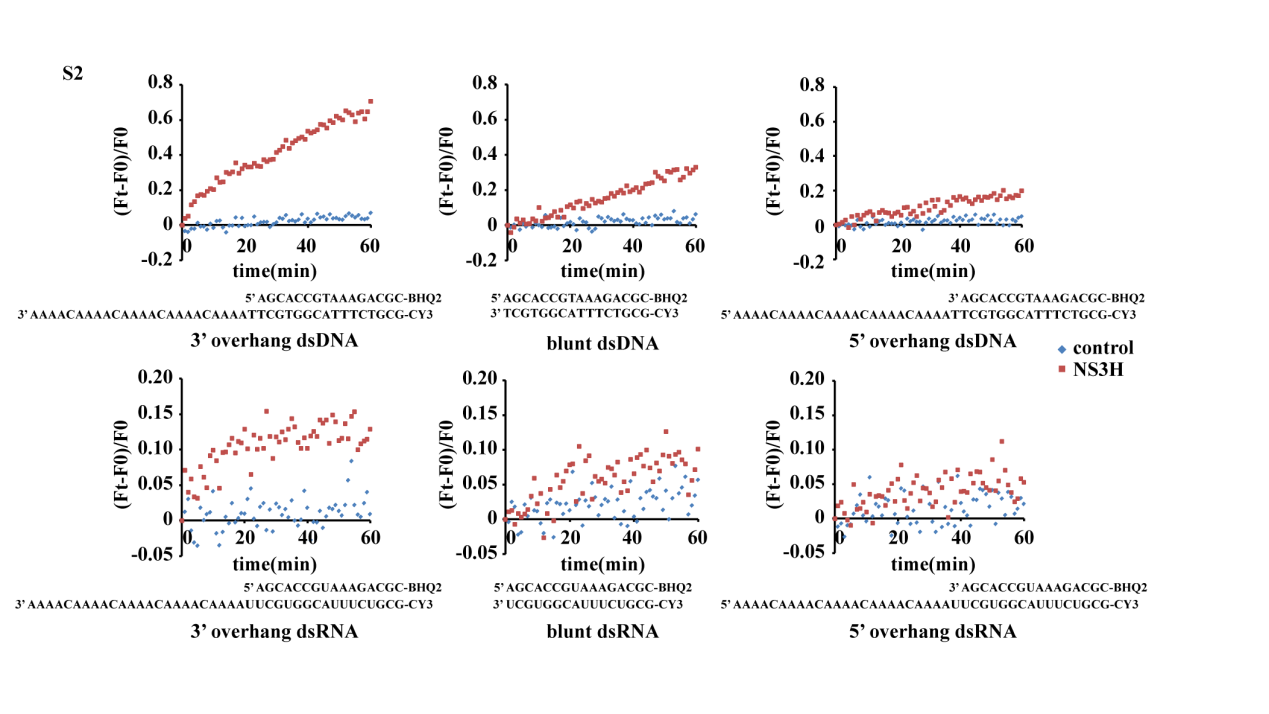


**Figure S2.** The real-time unwinding kinetics of ZIKV NS3H on 16 bp dsDNA (top) or 16 bp dsRNA (bottom) with a 3’ overhang, blunt ends or a 5’ overhang, respectively. 3 μM NS3H was used in the unwinding reactions. The fluorescent signal was measured every 1 min.


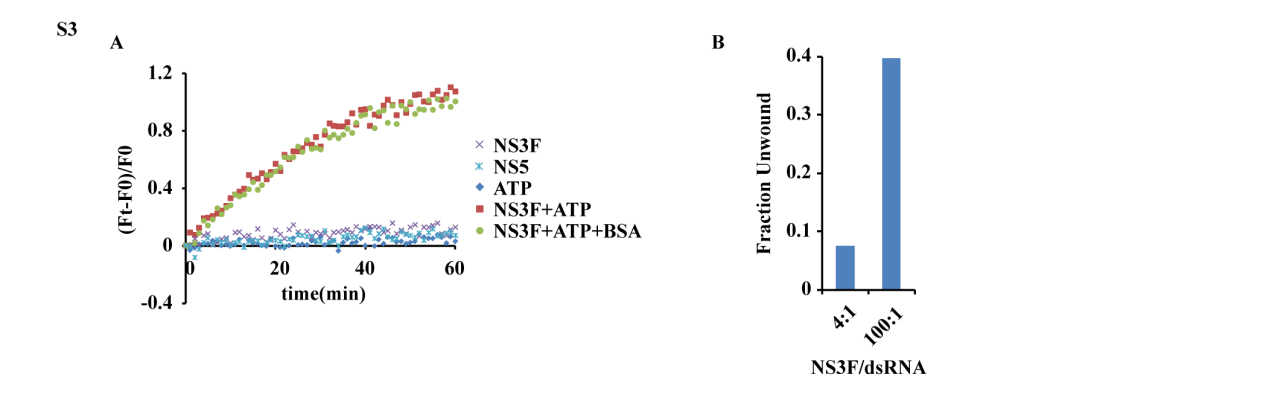


**Figure S3.** (A) dsRNA unwinding assay in the presence of NS3F (without ATP), NS5 (without ATP), ATP (without protein) , NS3F + ATP or NS3F + ATP + BSA. 200 nM NS3F or 100 nM NS5 was used in the reaction. (B) The unwinding fraction of NS3F under different helicase-to-substrate ratios in the presence of ATP at 60 min of the reaction. 16 bp dsRNA with 3’ overhang was used as substrate.


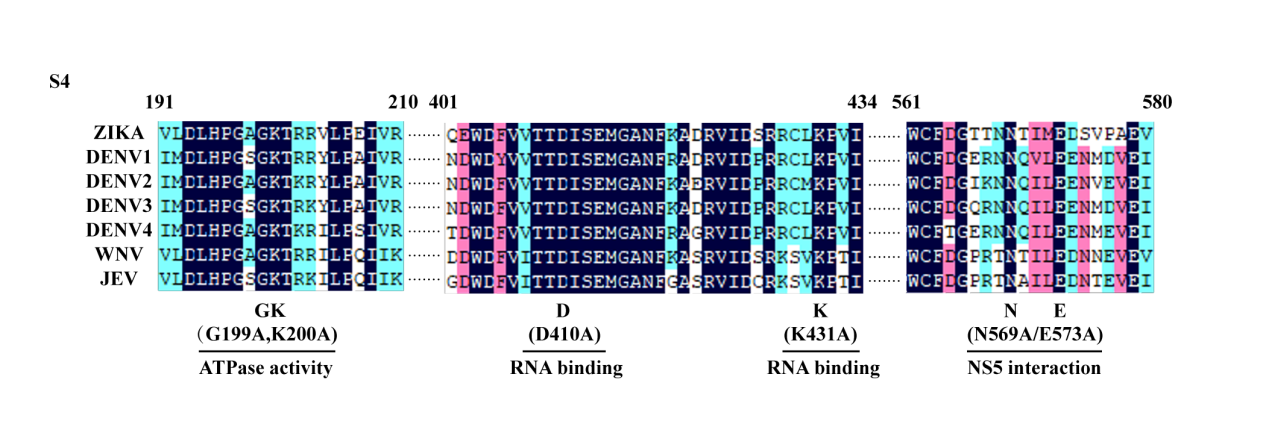


**Figure S4.** Amino acid sequence alignment of NS3 from multiple members of the flavivirus family. NS3 sequences of ZIKV, DENV-1, DENV-2, DENV-3, DENV-4, WNV and JEV are referred to GenBank accession numbers ARU07182.1, AHI43748.1, AOE23002.1, ANS59201.1, AAW51421.1, AHH02658.1 and AEO72439.1, respectively. Identical residues are shown in black. The mutated residues in this study are indicated at the bottom.


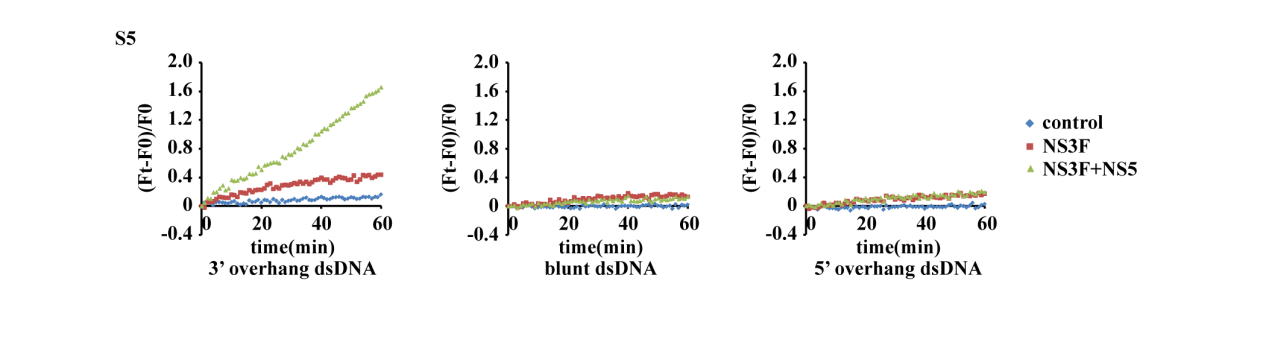


**Figure S5.** The unwinding kinetics of NS3F with or without NS5 on 16 bp dsDNA with a 3’overhang, blunt ends or a 5’ overhang. The unwinding reactions were performed with 100 nM NS3F and 100 nM NS5. Reactions without enzyme were used as controls. The fluorescent signal was measured every 1 min.


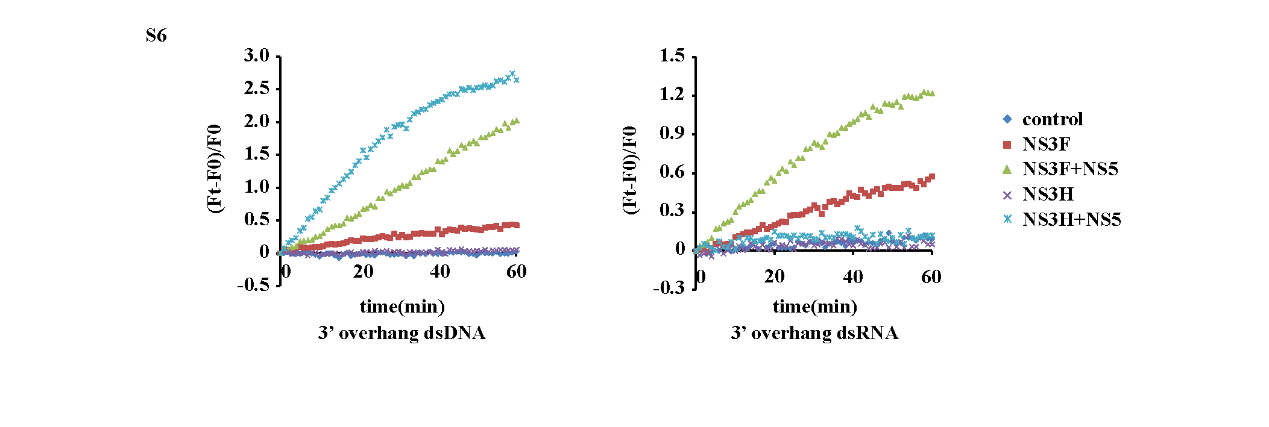


**Figure S6.** Effects of NS5 on the helicase activity of NS3F and NS3H in unwinding 16 bp dsDNA (left) or 16 bp dsRNA (right) with a 3’ overhang. The reactions were performed with 100 nM NS3F (or NS3H) and 100 nM NS5.


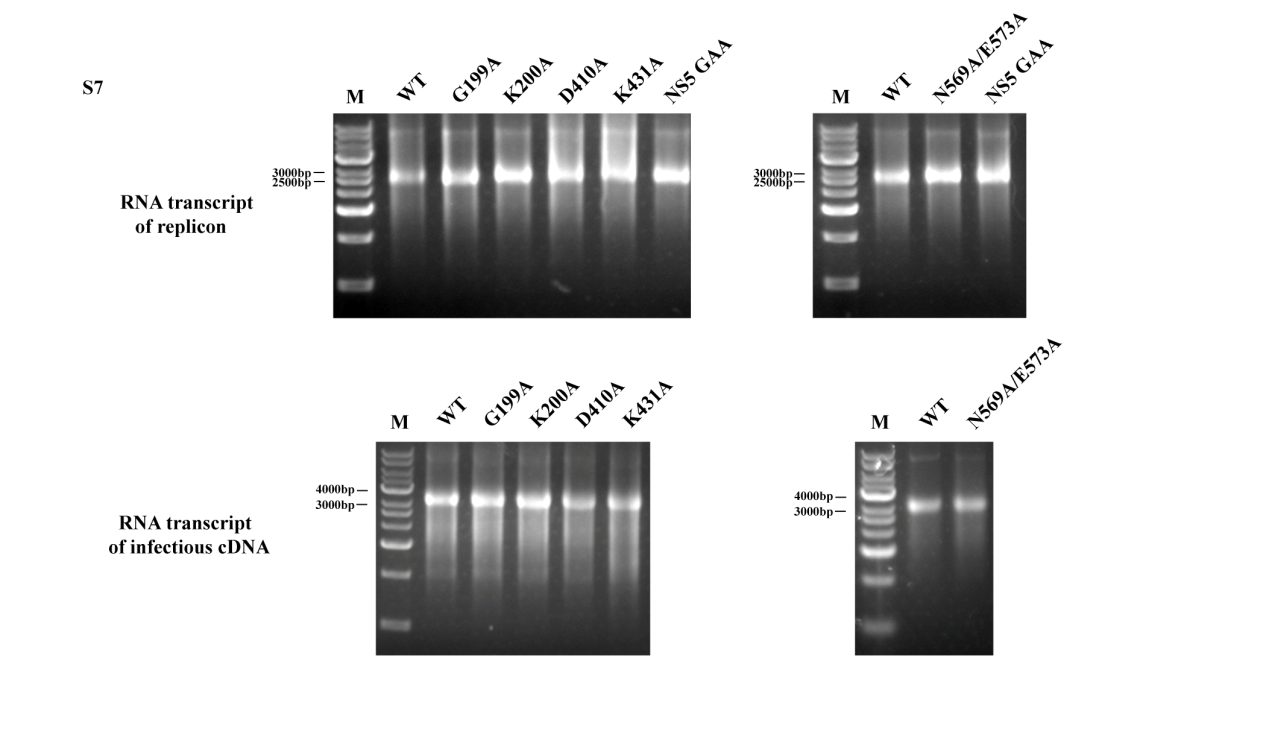


**Figure S7.** RNA transcribed *in vitro* from the ZIKV replicon (top panel) or infectious cDNA clone (bottom panel) was analyzed by 1% agarose gels.


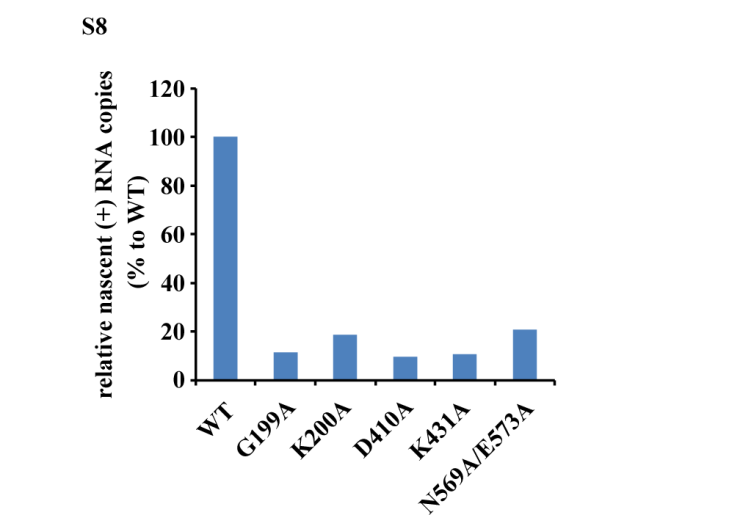


**Figure S8.** Newly transcribed viral RNA quantification. BHK cells were transfected with viral RNA derived from ZIKV infectious clones and treated by s^4^UTP for 3 h before RNA extraction at 9 h and 48 h post transfection. s^4^U-RNA was conjugated by MTS-biotin and isolated by streptavidin magnetic beads. Newly transcribed viral (+) strand RNA was detected by strand specific RT-qPCR. The data were expressed as relative newly transcribed (+) strand RNA amount at 48 h post transfection, normalized by total (+) strand RNA detected at 9 h as the input.
